# Recent Advances in Marine Biomaterials Tailored and Primed for the Treatment of Damaged Soft Tissues

**DOI:** 10.3390/md21120611

**Published:** 2023-11-25

**Authors:** Moon Sung Kang, Hyo Jung Jo, Hee Jeong Jang, Bongju Kim, Tae Gon Jung, Dong-Wook Han

**Affiliations:** 1 Department of Cogno-Mechatronics Engineering, Pusan National University, Busan 46241, Republic of Korea; mskang7909@gmail.com (M.S.K.); lisa024546@gmail.com (H.J.J.); h78crom@naver.com (H.J.J.); 2 Dental Life Science Research Institute/Innovation Research & Support Center for Dental Science, Seoul National University Dental Hospital, Seoul 03080, Republic of Korea; bjkim016@snu.ac.kr; 3 Medical Device Development Center, Osong Medical Innovation Foundation, Cheonju-si 28160, Republic of Korea; 4 Institute of Nano-Bio Convergence, Pusan National University, Busan 46241, Republic of Korea

**Keywords:** marine biomaterial, tissue engineering, regenerative medicine, scaffold, soft tissue

## Abstract

The inherent self-repair abilities of the body often fall short when it comes to addressing injuries in soft tissues like skin, nerves, and cartilage. Tissue engineering and regenerative medicine have concentrated their research efforts on creating natural biomaterials to overcome this intrinsic healing limitation. This comprehensive review delves into the advancement of such biomaterials using substances and components sourced from marine origins. These marine-derived materials offer a sustainable alternative to traditional mammal-derived sources, harnessing their advantageous biological traits including sustainability, scalability, reduced zoonotic disease risks, and fewer religious restrictions. The use of diverse engineering methodologies, ranging from nanoparticle engineering and decellularization to 3D bioprinting and electrospinning, has been employed to fabricate scaffolds based on marine biomaterials. Additionally, this review assesses the most promising aspects in this field while acknowledging existing constraints and outlining necessary future steps for advancement.

## 1. Introduction

Human soft tissues, encompassing intricate structures like skin, cartilage, and nerves, can undergo defects due to factors such as aging, trauma, infections, diseases, and tumors. Addressing these defects has involved techniques like xenografts, autografts, and allografts; however, each carries its own disadvantages, such as limited donor sites and disease transmission concerns [1,2,3]. While natural healing processes are effective in many cases, critical situations may exceed the body’s ability to restore both structure and function. Consequently, clinical approaches strive to replace damaged tissues with artificial prosthetics or grafts [4,5,6]. However, challenges persist including high costs, immunological reactions, and insufficient biochemical and structural similarity with the native extracellular matrix (ECM) [7,8,9]. Tackling these challenges through tissue engineering approaches necessitates biocompatible materials and biomimetic structures for repairing or replacing defective human soft tissues. Promising methods include cell-based therapies that manipulate cell biochemistry and engineered scaffolds, supporting cell attachment and proliferation. Tissue engineering scaffolds particularly provide appropriate characteristics such as pore size, shape, distribution, biodegradability, low immunogenicity, and non-cytotoxicity. Diverse materials like polymers, ceramics, metals, and composites have been used for scaffold fabrication. Despite their benefits, high production costs remain a limitation, prompting the exploration of natural materials as alternatives. The natural materials exhibit enhanced biocompatibility through improved cell surface interaction, fostering better cell attachment and growth.

On the other hand, the vast ocean, covering around 70% of the Earth’s surface and harboring 90–95% of biosphere volume, teems with biological diversity, offering abundant resources including skin, bone, and shells from marine organisms. These resources hold significant potential as components for marine biomedical materials. Recent studies have revealed that the ocean’s renewable and eco-friendly resources attract attention against current environmental challenges and energy shortages [10]. Biomaterials derived from aquatic species and their waste or byproducts are deemed renewable biosources, offering potential applications in biomedicine, pharmacology, and other fields due to their high volatility and energy efficiency [11,12]. Among various applications, the medical domain stands out, integrating marine-derived materials into modern medical advancements. These materials offer distinct benefits such as biocompatibility, biodegradability, and biological activity, driving their utilization in scaffold materials, wound dressings, and drug carriers for tissue engineering [13,14,15]. This emphasis on marine biomaterials has become a central theme in material research and development [16].

Among various applications, the medical domain stands out, integrating marine-derived materials into modern medical advancements. These materials offer distinct benefits such as biocompatibility, biodegradability, and biological activity, driving their utilization in scaffold materials, wound dressings, and drug carriers for tissue engineering. This emphasis on marine biomaterials has become a central theme in material research and development.

**Figure 1:**
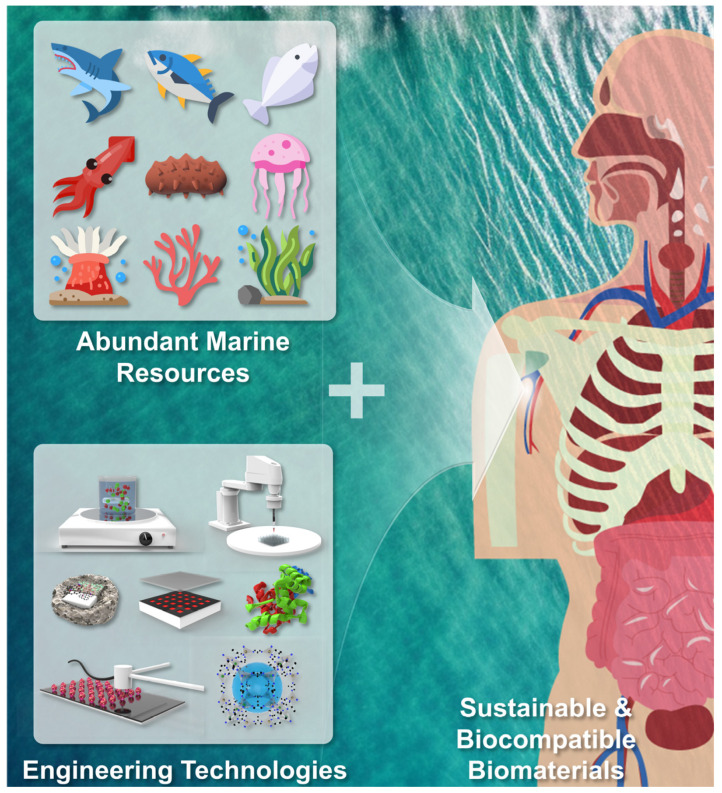
Marine biomaterials tailored and primed for treatment of damaged soft tissues.

In line with recent highlights in marine biomaterials for biomedical applications, we discuss the commonly used marine biomedical materials, classified as polysaccharides and proteins that are derived from various marine organisms, for their application in soft tissue engineering (Figure 1 and Table 1). This review presents a comprehensive overview of the extraction, processing techniques, and chemical and biological attributes of prevalent marine biomaterials. We propose that leveraging the distinctive biological characteristics of marine biomaterials has the potential to open up new avenues for tissue engineering technology.

## 2. Application of Marine Polysaccharides in Soft Tissue Engineering

Polysaccharides, with diverse functional groups including hydroxyl, amino, carboxylic acid, and aldehydes, offer excellent conjugation properties, making them suitable for applications in drug delivery, gene delivery, tissue engineering, and various biomedical uses due to their biodegradable, biocompatible, and hydrophilic nature (Table 2). Chitins are aminopolysaccharides, discovered in cuticles of various shellfishes, crustaceans (e.g., crabs and shrimps), as well as skeletons of see sponges (i.e., red sea sponges and demosponges), and have emerged as valuable biomaterials [17,18,19,20].

These sponges create three-dimensional skeletons composed of partially brominated α-chitin mineralized with silica and calcium carbonates [21]. The ability of Verongida sponges to grow rapidly under marine conditions makes sponge chitin a sustainable resource. These scaffolds are highly biocompatible, possess a unique design, and can be easily isolated, making them appealing for biomedical research. In the field of soft tissue engineering, they offer favorable properties for supporting cell attachment, proliferation, and differentiation, thus promoting the regeneration of damaged or diseased tissues [22,23].

**Figure 2:**
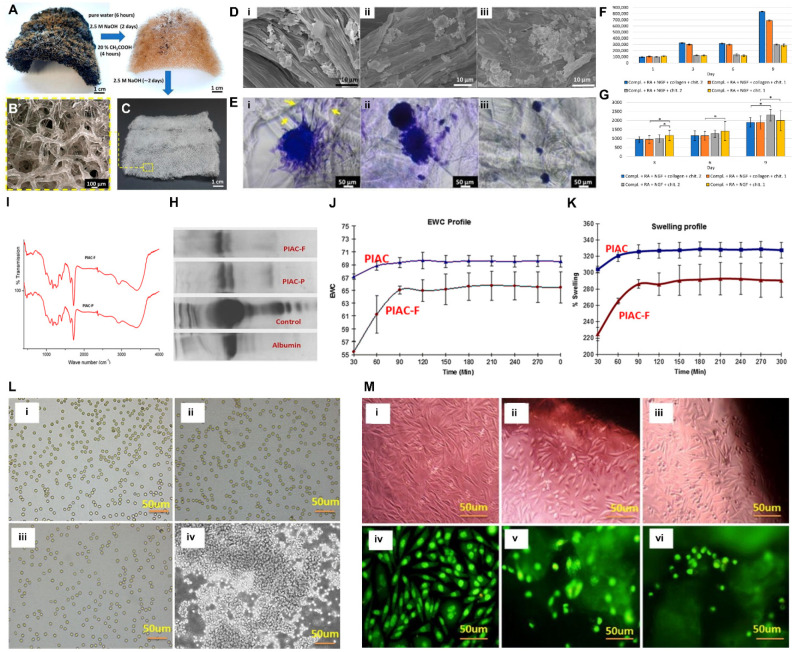
Marine polysaccharides in soft tissue engineering. (**A**–**G**) Deproteinized chitin for skin and neural tissue engineering. (**A**) Dried fragment of *A. fistularis* marine sponge. (**B**) SEM and (**C**) digital images of the deproteinized chitin scaffolds. (**D**) SEM images after 7-day in vitro culture of (**i**) BALB/3T3 cells, (**ii**) HDFs, and (**iii**) HaCaT cells. (**E**) Crystal Violet staining after 7-day in vitro culture of (**i**) BALB/3T3 cells, (**ii**) HDFs, and (**iii**) HaCaT cells. (**F**) Proliferation of SH-SY5Y cells on chitin scaffolds. Yellow arrows indicate extruded filopodia for cell spreading. (**G**) Proliferation of SH-SY5Y cells in the field of vision on the microphotographs of deproteinized chitin scaffolds. The asterisk indicate statistical significance between groups (*p* < 0.05). Data reproduced from [25]. Copyrights MDPI 2021. (H-M) PIAC hydrogels for soft tissue engineering applications. (**H**) Infra-red (IR) spectral analysis of PIAC hydrogels. (**I**) SDS-PAGE analysis of plasma protein adsorption. (**J**) EWC profile and (**K**) swelling profile of PIAC hydrogels. (**L**) red blood cell (RBC) aggregation studies on (**i**) PIAC-P, (**ii**) PIAC-F, (**iii**) saline, and (**iv**) polyethyleneimine. (**M**) Cytocompatibility evaluations of L929 cells in the hydrogels: (**i**,**iv**) control, (**ii**,**v**) PIAC-P, and (**iii**,**vi**) PIAC-F hydrogel. Data reproduced from [29]. Copyrights Springer Nature 2015.

Mutsenko et al. fabricated ready-to-use tissue-engineered products using chitinous scaffolds from the marine demosponge *Ianthella basta* for cartilage tissue engineering [24]. They used an alkali-based procedure to isolate the scaffolds, effectively removing toxic components while maintaining the chemical composition, three-dimensional (3D) structure, and bioactivity. The resulting scaffolds exhibited natural interconnected porosity, which is crucial for cell infiltration and nutrient transport. To assess biocompatibility, they cultured human mesenchymal stem cells (hMSCs) on the scaffolds and observed that the cells adhered and proliferated well, maintaining their characteristic morphology. Moreover, the hMSCs demonstrated the ability to differentiate into adipocytes when cultured on the chitin scaffolds. Cryopreservation experiments revealed that chitin scaffolds can withstand the freezing process without significant structural or chemical changes. However, there was a reduction in cell viability and attachment after thawing, indicating that the cryopreservation of cells within the 3D scaffold is more challenging compared to traditional suspension cryopreservation. Cryomicroscopy suggested that ice formation within the chitin fibrils and subsequent ice propagation could contribute to cell damage during cryopreservation. In a similar study conducted by Machalowski et al., the authors fabricated deproteinized chitin scaffolds derived from Porifera (*Aplysina fistularis*) fir skin tissue engineering application [25] (Figure 2A–G). To fabricate the effect of chitin-based scaffolds, they require specific 3D geometry, porosity, and surface characteristics, and mechanical properties, and their nanoscale fibrous morphology should accelerate wound healing and provide antibacterial properties, yet variations in the properties and source origin impact its suitability for wound treatment applications [23,26]. The prepared marine sponge scaffolds exhibited characteristic macro-porous structures with interconnected pores of varying sizes. The degree of acetylation was calculated as 79%, indicative of chitin isolated through alkali treatment. It was found that there was a low degree of cytotoxicity in BALB/3T3 cell cultures, showing that the scaffold was non-toxic. It was observed that various cell types, including human dermal fibroblasts (HDFs), HaCaT keratinocytes, and SH-SY5Y neuronal cell lines, adhered and proliferated on the scaffold. HaCaT cells formed cohesive structures on the scaffold’s surface, mimicking the organization of skin tissue, with the presence of desmosomes. SH-SY5Y cells also demonstrated adherence and migration on the scaffold, suggesting its potential for tissue engineering in skin and neuronal regeneration. On the other hand, the future challenges include the necessity for optimization for highly biocompatible 3D cell cultures with cost-effective scaffold production and leveraging the scaffold’s attractiveness for neuronal cell migration.

The heteropolysaccharide alginate (Alg), derived from brown sea algae, consists of interconnected β-D-mannuronic (M) and α-L-guluronic (G) monomers [27]. Alg has been utilized as an ECM substitute in soft tissue engineering applications because of its high water retention and minimal toxicity [28]. Radhakrishnan et al. designed composite scaffolds composed of marine-derived heteropolysaccharides (i.e., Alg and chitosan), with the additional incorporation of polyethylene glycol (PEG) (namely, PIAC) through the condensation reactions [29] (Figure 2H–M). The partially crosslinked hydrogel (PIAC-P) was formed using chelation with Ca^2+^ ions, and the fully crosslinked hydrogel (PIAC-F) was achieved using crosslinking with glutaraldehyde. Water absorption profiles showed equilibrium swelling values, demonstrating their water content and holding capacity, making them suitable for cell growth and ECM deposition. The hydrogels exhibited porosity, with PIAC-F having slightly lower porosity due to glutaraldehyde crosslinking, but both maintained similar bulk density. Hemocompatibility tests showed negligible hemolysis and no adverse effects on blood fluidity. The PIAC hydrogels adsorbed total plasma proteins, particularly albumin, enhancing their biocompatibility. Cell compatibility assays, including MTT and live/dead assays, confirmed their cytocompatibility, with more than 95% cell viability, non-apoptotic cells, and healthy cell morphology on the hydrogel surfaces. The study suggested that PIAC-based hydrogels have the potential as ECM mimics for an L929 fibroblast cell line culture. For future works, optimizing the synthesis process would be required to achieve the desired properties and stability, balancing the mechanical properties, and controlling the extent of crosslinking to avoid cracking and breaking of hydrogels.

Kappa-carrageenan (κCA) is a naturally occurring linear water-soluble polysaccharide that contains a single sulfate group in each disaccharide unit, making up about 25 to 30% of its sulfate ester content. This biopolymer is mainly derived from red algae and well suited for use in tissue engineering due to its ability to mimic biological properties and its resemblance to the structures found in natural glycosaminoglycans (GAGs) [30,31,32]. Tavakoli et al. fabricated a sprayable and injectable hydrogel using visible-light crosslinked methacrylated κCA (KaMA) hydrogel (derived from brown algae) with the aim of applying it in wound-healing application [33]. Lower elastic and compressive moduli of 4 wt% highly methacrylated (4-HM) KaMA hydrogel led to greater bovine serum albumin (BSA) adsorption, which would be favorable for cell growth. In vitro experiments revealed that 4-HM KaMA hydrogels were suitable for use as sprayable/injectable biomaterials, with high cell viability (98 ± 2%). The metabolic activity of cells, as assessed with the MTT assay, indicated that 4-HM KaMA hydrogel types supported cell survival and proliferation, mainly due to exceptional mechanical properties affecting protein adsorption. Therefore, it is highlighted that the 4-HM KaMA hydrogel would be promising biomaterial for in situ wound dressings, emphasizing the use of visible-light initiators for safer cytocompatibility. However, understanding the factors contributing to differences in cell viability between 4-HM and 6-LM hydrogel samples would be needed for wider application in tissue engineering.

Tytgat et al. fabricated red-algae-derived κCA and gelatin blend hydrogel for adipose tissue engineering [34]. Two distinctive hydrogels, methacrylamide-modified gelatin (GEL-MOD) and methacrylate-modified κCA (CAR-MOD), were blended (GEL-MOD/CAR-MOD). In the in vitro biological evaluation, adipose-derived stem cells (ASCs) were seeded onto the GEL-MOD/CAR-MOD hydrogels, and they exhibited a high cell viability and proliferation rate within 7 days. CAR-MOD hydrogels exhibited spherical cell morphology, indicating non-adherent characteristics. In contrast, GEL-MOD hydrogels and their blends with CAR-MOD promoted ASCs’ attachment and displayed elongated cell shapes due to the presence of cell-interactive Arginylglycylaspartic acid (RGD) sequences in GEL-MOD. MTT assays revealed that CAR-MOD hydrogels had significantly lower cell viability after 1 day, attributed to the absence of cell-responsive moieties in κCA. The proliferation of attached cells increased with higher GEL-MOD concentration. The blended GEL-MOD/CAR-MOD had more viable cells compared to individual GEL-MOD and CAR-MOD, suggesting their potential for SC engineering applications. On the other hand, the weak and brittle structure of κCA under physiological conditions is one of the major concerns for utilization of κCA in biomedical engineering [35]. To address this issue, İlhan et al. proposed a novel approach using microwave-assisted methacrylation (Mw-κCa-MA) to functionalize κCar and investigated their utilization as bioink for 3D bioprinting of cartilages [36]. Live/dead assays showed high cell viability in Mw-κCa-MA hydrogels (90% on day 1). Scanning electron microscopy (SEM) revealed that cells in Mw-κCa-MA hydrogels displayed a round chondrocyte morphology on day 7. A histological analysis demonstrated promoted ECM deposition in Mw-κCa-MA hydrogels and a round cartilage morphology with lacuna formation. An immunocytochemical analysis confirmed more staining for collagen (Col) type II and aggrecan (ACAN) in Mw-κCa-MA hydrogels. GAG content and Col content were significantly increased in Mw-κCa-MA hydrogels at 21 days of the culture. Overall, microwave-assisted methacrylation resulted in improved cell viability, chondrogenic differentiation, and ECM deposition in κCar-MA hydrogels, which could be applied for future bioprinting of cartilages. Further study could address the limitations of the conventional methacrylation method used, resulting in a low degree of methacrylation in κCa and unstable hydrogels, as well as balancing material strength and viscosity for 3D printing. Future works can involve the exploration of microwave-assisted methacrylation for enhanced κCa methacrylation and extended cell culture studies to assess long-term effects.

Fucoidans, which are polysaccharides found in brown seaweed and some marine invertebrates, have been extensively studied over the past decade due to their diverse biological activities [37]. These activities include anticoagulant and antithrombotic effects, antiviral properties, anti-tumor and immunomodulatory functions, anti-inflammatory responses, reduction in blood lipids, antioxidant capabilities, and anti-complementary actions [38,39,40]. Fucoidans also demonstrate potential in addressing various health conditions and therapeutic applications, with the added advantage of being readily available from affordable sources [41]. Huang et al. developed biodegradable chitosan (CS)–fucoidan nanoparticles (CS–F NPs), which were designed to carry basic fibroblast growth factors (bFGFs) for neural tissue engineering applications [42]. Higher ratios of CS to fucoidan within the NPs corresponded to increased cell survival rates indicating no cytotoxic effects even at a concentration of 125 ng/mL. In addition, bFGF released from CS-F NPs effectively promoted neurite extension, which exhibited comparable results in nerve growth factor (NGF)-treated groups. Notably, a lower cumulative amount of bFGF in the CS-F NPs can be attributed to the presence of fucoidan, which reduced the required amount of bFGF for promoting neurite extension. This effect could be analogous to the role of heparin in enhancing bFGF functionality. Heparin sulfate (HS), similar in structure to heparin, is known to be an essential co-factor in FGF signaling processes, suggesting that fucoidan may serve a similar function in enhancing bFGF’s impact on neurite growth and FGF signaling processes. Fine-tuning the CS-F NPs to ensure precise control over the release kinetics and therapeutic efficacy of bFGF would be beneficial for effective nerve tissue engineering. Furthermore, elucidating interaction between fucoidan and bFGF activity could establish fucoidan as a highly prospective biomaterial for applications in nerve tissue engineering. On the other hand, Martins et al. utilized brown-algae-obtained marine laminarin microparticles functionalized with a methacrylate moiety and loaded with platelet lysates (PLs) (namely, MeLam microparticles) for L929 fibroblasts’ culture [43]. The MeLam microparticles feature methacrylate groups on a photosensitive laminarin backbone, serving as anchor points for thiol-biotin and biotinylated RGD molecules to enhance cell adhesion. The MeLam microparticles could serve as a controlled delivery system for various growth factors within PL, promoting cell adhesion and growth. The dual release of vascular endothelial growth factor (VEGF) and transforming growth factor-β1 (TGF-β1) was observed, potentially aiding in cell proliferation and tissue construct formation. Functionalization with biotin-RGD molecules enhanced cell adhesion, and L929 fibroblasts cultured on PL-loaded microparticles showed increased cell attachment, morphology changes, and higher viability. These results suggest that the developed MeLam microparticles have a positive impact on cell proliferation and can be provided as ideal platforms for 3D soft tissue construct formation. Further study could assess laminarin‘s interactions with bioactive factors and controlling its release rates from MeLam microparticles.

Ulvans, a distinct group of water-soluble sulfate polysaccharides derived from the cell walls of Ulva seaweeds, are primarily composed of sulfate, rhamnose, and xylose, and iduronic and glucuronic acids [44]. These ulvans have a chemical similarity to GAGs and offer significant antioxidant and immunomodulatory characteristics, rendering them well suited for various applications, including their use in wound dressings [45]. Madub et al. extracted cellulose from Ulva seaweeds and fabricated electrospun matrices in combination with polydioxanone (PDX), poly-l-lactide (PLLA), and poly-DL-lactide (PDLLA) for potential applications in nanofibrous scaffolds [46]. Ulvan–cellulose has been successfully extracted and thoroughly characterized, then converted into electrospun nanofibrous matrices as well as in combination with PLLA, PDLLA, and PDX. The addition of these materials did not significantly alter the physicochemical properties of cellulose. In vitro, ulvan–cellulose demonstrated favorable interactions with fibroblast cells, resulting in the formation of actin filaments, filopodial, and lamelipodial protrusions. In vivo studies with Wistar rats indicated positive tissue–material interactions, characterized by a low foreign body response and strong neovascularization. Based on the results, ulvan–cellulose is suggested to have significant potential to improve diabetic wound-healing applications. Furthermore, promising results were shown regarding cell–matrix interactions and in vivo biocompatibility of ulvan-based matrices, particularly in terms of fibrous capsule thickness and neovascularization. The Australian ulvacean macroalgae species produces a distinct type of ulvan (Ul84) with exceptional characteristics, including a high molecular weight of approximately 700 kDa, natural sulfation, a significant presence of rhamnose units recognized by cell membrane lectins (around 50%), as well as the iduronic and glucuronic acid components, which resemble mammalian GAGs such as dermatan sulfate and heparan sulfate [47]. Chen et al. reported methacrylated Ul84 (UlMA)-incorporated GelMA (UlMA-GelMA) bioink for skin tissue engineering applications [48]. The study examined the printability, mechanical stiffness, in vitro cellular growth, ECM production, and in vitro degradation of UlMA-GelMA bioinks. The bioinks demonstrated suitable printability at different temperatures, and their geometric fidelity remained intact during 3D printing. The mechanical stiffness of the hydrogels increased with higher UlMA content, enhancing their suitability for skin tissue engineering. The HDF-laden structures exhibited good cell viability, proliferation, and ECM production, indicating their cytocompatibility. UlMA incorporation influenced gene expression, particularly in the down-regulation of the COL3A1 gene. The printed structures were also able to deposit key ECM proteins such as Col I, Col III, elastin, and fibronectin. In vitro degradation studies showed that the UlMA-containing structures had reduced porosity and increased resistance to enzymatic degradation. These findings suggest that UlMA-GelMA bioinks have potential in skin tissue engineering, wound healing, and ECM modulation. Further investigation into the role of ulvan in gene expression regulation during the wound-healing process would help with exploiting the potential of ulvan in tissue engineering.

**Figure 3:**
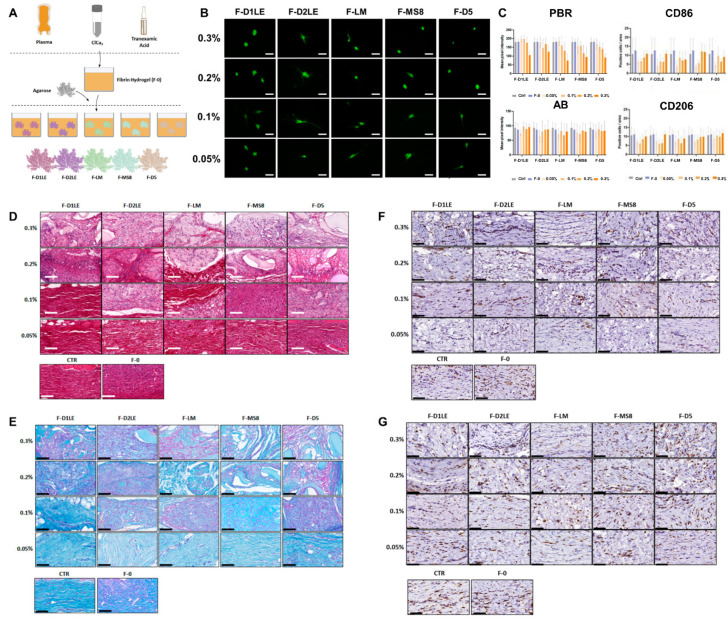
Marine-derived polysaccharide hydrogel obtained from different agarose sources. (**A**) Schematic diagram of FA hydrogel preparation. (**B**) Live/dead assay on HDFs cultured in different FA hydrogel groups. (**C**) In vivo expression of ECM remodeling markers based on immunohistochemical analysis using Wistar rat models. Immunohistochemical images of (**D**) picrosirius red marking Col, (**E**) alcian blue staining GAGs and glycoproteins, (**F**) H&E-stained image with CD86 (brown), and (**G**) H&E-stained image with CD206 (brown). Data reproduced from [52]. Copyrights MDPI 2023.

Agarose is a polysaccharide derived from marine sources, known for its long, non-toxic, and purified molecular chains with minimal impurities, low degradability, and characteristics such as transparency, consistent gel strength, and thermal reversibility, making it suitable for various applications, especially in hydrogel formation [49]. To address agarose’s low biofunctionalities, it is often combined with other biomaterials like CS, graphene oxide (GO), silk fibroin (SF), Col, and fibrin for specific applications [50,51,52]. Especially fibrin–agarose (FA) biomaterials have been applied successfully to create various tissues and organs, demonstrating improved biomechanical properties, biocompatibility, and in vivo safety for potential patient use [53]. Ortiz-Arrabal et al. synthesized FA scaffolds using five different types of agarose sources (referred to as F-D1LE, F-D2LE, F-LM, F-MS8, and F-D5) for soft tissue engineering applications [54] (Figure 3). An ex vivo analysis indicated high biocompatibility of all FA scaffolds with HDFs. Biomechanical properties were influenced by the type and concentration of agarose, with an increased Young’s modulus in most FA scaffolds compared to fibrin alone. In vivo studies in rats showed no systemic effects on hematological parameters and no histological alterations in vital organs. Macroscopic and histological assessments confirmed the integration of FA scaffolds at the implant site. The biodegradation rate varied with agarose type and concentration, with higher concentrations demonstrating better maintenance. Histochemical and immunohistochemical analyses revealed variable amounts of Col, proteoglycans, and expression of matrix metallopeptidase 14 (MMP14) in FA scaffolds. M1 type and M2 type macrophages were present in similar quantities across all samples. These findings support the biocompatibility and potential clinical use of FA scaffolds in tissue engineering, with the option to select specific agarose types and concentrations for precise biomechanical properties and in vivo reabsorption times. Future work should focus on optimizing biomechanical properties of these agarose-based biomaterials based on the type and concentration of agarose used, to offer more opportunities for clinical uses.

On the other hand, marine prokaryotes produce a wide range of polysaccharides with unique structures and intriguing biological characteristics [55]. These naturally occurring polysaccharides, similar to GAGs, can be tailored through simple and reproducible chemical processes, making them potentially valuable for medical applications [56]. Among these marine prokaryotes, *Alteromonas infernus* stands out for producing GY785, a distinctive high-molecular-weight exopolysaccharide featuring low-molecular-weight derivatives; resembling heparin; exhibiting anticoagulant properties; and having recently been found to promote the chondrogenic differentiation of adipose-derived stromal cells [57]. Rederstorff et al. proposed combining the branched high-molecular-weight polysaccharide GY785 with silated hydroxypropyl methylcellulose hydrogel (Si-HPMC) scaffolds to create a hydrogel suitable for cartilage tissue engineering, possessing both biological and biomechanical competence [58]. The prepared Si-HPMC/GY785 hydrogels displayed specific interactions with chondrogenic growth factors, TGF-β1, and bone morphogenic protein-2 (BMP-2), suggesting their suitability as a platform for delivering bioactive agents. Cytocompatibility studies revealed that they did not exhibit cytotoxic effects, supporting their safety for use in tissue engineering applications. In 3D cultures, Si-HPMC/GY785 hydrogels maintained a differentiated chondrocyte-like phenotype, characterized by increased expression of chondrogenic markers (Col II and ACAN) and reduced expression of the dedifferentiation marker (Col I) in comparison to 2D cultures. Dedifferentiated chondrocytes regained their chondrocytic phenotype when placed in a 3D culture within these hydrogels. Furthermore, in vivo experiments demonstrated the formation of cartilaginous tissue containing GAGs, ACAN, and Col II, while Col I remained minimal. This suggests that Si-HPMC/GY785 hydrogels have the potential for effective cartilage tissue engineering, maintaining the chondrocyte viability and phenotype, and promoting tissue formation in an animal model. Critical for the successful creation of functional cartilage tissue is the assurance of keeping high cell viability and differentiation capacity within the Si-HPMC/GY785 hydrogel. Additionally, conducting thorough in vivo studies is essential in models closely resembling articular cartilage defects, where endogenous growth factors are abundant. In another study by Rederstorff et al., two distinctive marine exopolysaccharides (HE800 and GY785) were incorporated into Si-HPMC hydrogel for cartilage tissue engineering [59]. HE800 is a high-molecular-weight linear polysaccharide, which consists of a repeating tetrasaccharide unit and includes two glucuronic acid units, one N-acetyl-glucosamine, and one N-acetyl-galactosamine [60]. In a 2D in vitro culture, the Si-HPMC hydrogel compositions did not exhibit cytotoxicity, with no adverse effects on cell viability. However, in a 3D culture, the Si-HPMC hydrogels containing 0.67% HE800 significantly reduced the viability of MC3T3-E1 and C28/I2 cells, particularly at this concentration. The presence of HE800, hyaluronic acid (HA), or GY785 within the hydrogels encouraged cell attachment, and the dispersion of cells improved with increased concentrations of these polysaccharides. In 3D cultures, C28/I2 cells formed clusters of varying morphology, with larger clusters observed in the presence of HA or GY785. MC3T3-E1 cells retained a round shape in hydrogels without HA or GY785, while the presence of GY785 alone resulted in alcian blue staining, suggesting GAG production. Overall, these findings indicate that the Si-HPMC hydrogels can influence cell viability, attachment, and spatial organization in both 2D and 3D culture settings, with differential effects of HE800 and GY785 on various cell types. Further research for optimizing Si-HPMC hydrogel not to negatively impact its gelation, pH, or osmolarity in the culture environment could explore their utility in creating patches or other constructs for cartilage regeneration.

In summary, the vast range of structural and functional options offered by polysaccharides, coupled with their abundance and renewability in sources, has led to their utilization in numerous soft tissue engineering applications. Marine-derived polysaccharides such as chitin, chitosan, κCA, fucoidans, ulvan, agarose, and the prokaryote GY785 have displayed notable biocompatibility and biodegradability. Their resemblance to native ECMs facilitates desired cellular responses such as attachment, proliferation, and differentiation into specific cell types. However, to fully utilize the potential of these marine polysaccharides in tissue engineering, several challenges should be addressed. These encompass addressing issues including limited cell viability after cryopreservation, the variable suitability of polysaccharides from different sources, enhancing the mechanical properties and stability of polysaccharide-based hydrogels, and optimizing their biodegradability. Each accomplishment can open avenues for refining existing biomaterials and solving limitations for advancing soft tissue engineering applications.

**Table 2:** Marine polysaccharides in soft tissue engineering.

Engineered Forms	Applications	Marine Biomaterials	Sources	Cells/Animal Tested	Key Findings	Ref.
Acidic isolated chitin	Cartilage	Chitin	Porifera (*Lanthella basta*)	Human mesenchymal stromal cells	Cell viability and adipogenic potential were retained after cryopreservation.	[24]
Deproteinized chitin	Skin and neural tissues	Chitin	Porifera (*Aplysina fistularis*)	- BALB/3T3 cells, - HDF - HaCaT cells - SH-SY5Y cells	Skin and neural cells exhibited inherent phenotypes on the scaffolds.	[25]
PIAC hydrogel	Skin	- Alginate - Chitosan	- Brown algae for alginate	L929 fibroblast	The prepared hydrogel provided fibroblasts favorable for ECM-mimicking 3D microenvironment.	[29]
KaMA hydrogel	Skin	- κCa	- Red algae	- HeLa cells - L929 cells	Injectable and sprayable KaMA hydrogel supports survival and growth of skin cells.	[33]
GEL-MOD/CAR-MOD hydrogel film	SC engineering	- κCa	- Red algae	ASCs	GEL-MOD/CAR-MOD hybrid hydrogel supported viability and proliferation of ASCs.	[34]
Bioprinted Mw-κCa-MA hydrogel	Cartilage	- κCa	- Red algae	ATDC5 cell	Microwave-assisted methacrylation resulted in improved cell viability, chondrogenic differentiation, and ECM deposition in κCa-MA hydrogels.	[35]
CS-F NP	Nerve	Fucoidan	Brown seaweed (*Fucus vesiculosus*)	PC12 cell	CS-F NPs exhibited sustained bFGF release to induce neurogenic differentiation of PC12 cells.	[42]
MeLam microparticle	Skin	Laminarin	Brown algae	L929 cell	Encapsulated PL promoted the enhancement of L929 cell adhesion and proliferation.	[43]
Cellulose-PLLA, PDLLA, and PDX electrospun nanofiber	Skin	Ulvan	Green seaweed	- L929 cell - Wistar albino rats	Ulvan cellulose accelerated fibroblasts’ growth and in vivo angiogenesis.	[46]
Bioprinted UlMA-GelMA hydrogel	Skin	Ulvan	Ul-84	- HDFs	Ul84 supported fibroblast growth and deposition of key dermal ECM components.	[48]
FA hydrogel	Skin	Agarose	Red algae (*Gracilaria and Gelidium*)	- HDFs - Wistar rats	FA hydrogel showed excellent biocompatibility with pro-regenerative process with M2-type CD206-positive macrophages.	[54]
Si-HPMC/GY785 hydrogel	Cartilage	GY785	Deep-sea bacteria (*Alteromonas infernus*)	RACs Swiss nude mice	Si-HPMC/GY785 facilitated chondrogenesis through interactions with growth factors, gene expression, and in vivo cartilage-like ECM formation.	[58]
HE800/GY785-Si-HPMChydrogel	Cartilage	HE800 and GY785	NA	MC3T3-E1 cells C28/I2 cells	Addition of HE800 and GY785 in Si-HPMC hydrogel increased the mechanical properties and induced polysaccharide-specific effects on cell viability and spatial organization.	[59]

## 3. Application of Marine Proteins in Soft Tissue Engineering

Traditional sources of collagen extraction from bovine and porcine origins have faced limitations in recent years due to dietary restrictions, religious constraints, and concerns related to diseases like bovine spongiform encephalopathy (BSE), transmissible spongiform encephalopathy (TSE), and foot-and-mouth disease (FMD) [61]. As an alternative, marine organisms have gained attention as promising sources of proteins including collagen (Table 3). These organisms, including fish and sea urchin wastes, undersized fish, and by-catch creatures like jellyfish, sharks, starfish, and sponges, are considered viable sources of collagen with no religious- or disease-related limitations [62,63,64]. Using discarded and underutilized marine biomass can contribute to a sustainable approach for collagen extraction while minimizing environmental impact [65]. Especially Spongin, a halogenated scleroprotein found in the mineral-free skeletons of demosponges, particularly those in the subclass keratosa (*Chelonaplysilla violacea*), constitutes the fibrous structure of bath sponges that have been utilized mainly for bone tissue engineering [66]. In addition, *Chondrosia reniformis*, a demosponge, is an abundant source for marine collagen, exhibiting suitability for medical, surgical, and cosmetic applications, with recent interest focusing on its use as an organic template for silicification, enhancing the mechanical properties of biomimetically inspired hybrid xerogels [67].

**Figure 4:**
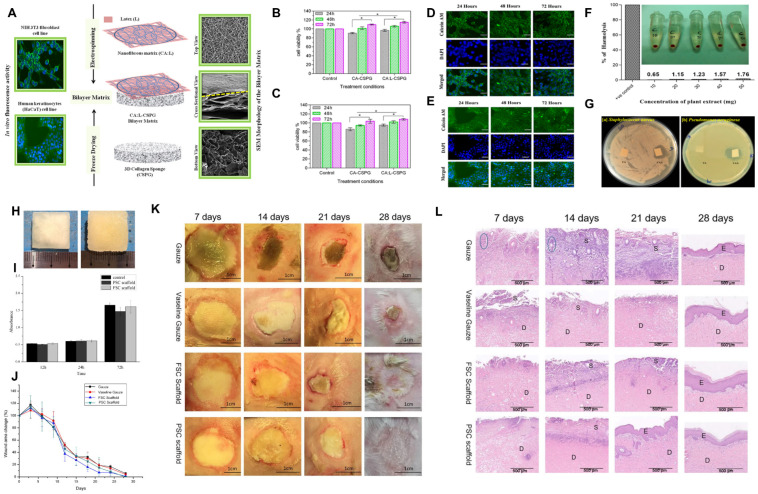
Marine proteins in soft tissue engineering. (**A**–**G**) Bilayered CA:L-CSPG nanofibrous sponge for wound-healing applications. (**A**) Schematic diagram of CA:L-CSPG nanofibrous spongy fabrication with NIT3T3 and HaCaT cell cultures. Viability of (**B**) NIT3T3 cells and (**C**) HaCaT cells within CA-CSPG and CA:L-CSPG sponge. The asterisks denote statistical significance (*p* < 0.05). Immunocytochemical analysis on (**D**) NIT3T3 cells and (**E**) HaCaT cells within CA:L-CSPG sponge. (**F**) Hemolytic activity of the latex. (**G**) Antimicrobial activity of the CA:L-CSPG sponge using (left) *S. aureus* and (right) *P. aeruginosa.* Data reproduced from [68]. Copyright Aerical Chemical Society 2020. (**H**–**L**) Freeze-dried FSC scaffolds for burn wound dressing application. (**H**) Digital images of (left) PSC and (right) FSC scaffolds. (**J**) Wound closure area and (**K**) burn wounds in gauze, Vaseline gauze, and FSC and PSC scaffolds. (**L**) H&E-stained microscopic sections in gauze, Vaseline gauze, and FSC and PSC scaffolds. The blue circle indicates blisters. The S, D, and E represent inflammatory cells, dermis, and epidermis, respectively. Data reproduced from [69]. Copyrights Oxford Academic 2020.

Ramanathan et al. introduced cellulose acetate (CA)-Col bilayer nanofibrous spongy dressing matrices for wound-healing application [68] (Figure 4A–G). CA and Col were obtained from *Arothron stellatus*. The bioactive latex from *Calotropis procera* was incorporated via electrospinning into CA nanofiber to produce a biohybrid nanomaterial (CA:L) that was deposited onto a bilayer 3D collageneous porous sponge (CSPG) to be used as a bilayer wound dressing material (CA:L-CSPG). Both the NIH 3T3 fibroblast and HaCaT keratinocytes showed increased cell viability when seeded on the nanofibrous matrix side of the bilayer scaffold, with CA:L-CSPG demonstrating significantly high cell viability and proliferation. Scanning electron microscopy confirmed uniform cell distribution and excellent biocompatibility on the CA:L-CSPG nanofibrous layer. Additionally, the CA:L-CSPG membrane exhibited strong antioxidant activity, no hemolysis, and significant antibacterial activity against both Gram-positive and Gram-negative bacteria. The antimicrobial and hemocompatible nature of the bioactive latex opens avenues for developing wound-healing materials; however, further approaches to control the initial burst release of bioactive molecules from the CA:L-CSPG membrane would be needed to prevent bacterial infections at the wound site. Shi et al. compared the burn-wound-healing effects of two Col porous sponge scaffolds obtained from porcine or marine sources [69] (Figure 4H–L). Both porcine-skin-derived Col (PSC) and fish-scale-derived Col (FSC) were freeze-dried to prepare the porous scaffolds. The appearance and porosity of PSC and FSC scaffolds were evaluated, showing high porosity suitable for cell in-growth and nutrient exchange. The composition analysis confirmed that both materials were primarily Col I. Water uptake and vapor transmission properties were examined, with both scaffolds demonstrating acceptable water-holding capacity and vapor transmission rates, important for wound healing. Cytotoxicity tests on L929 cells showed acceptable toxicity levels for both PSC and FSC scaffolds. In a visual comparison of burn wound healing, both PSC and FSC scaffolds outperformed traditional gauze and Vaseline gauze. The healing process was quicker, and hair growth was observed in the scaffold-treated groups. Histological examinations confirmed accelerated wound recovery with less inflammation in the scaffold groups, with PSC scaffolds showing the fastest re-epithelization. Based on these results, it is suggested that marine Col has comparable biofunctionality with porcine Col to enhance the wound-healing process. Future perspectives include exploring FSC’s potential application in other chronic wounds and endowing it with additional properties such as mechanical integrity and antibacterial properties.

Sharks, unlike humans and most other vertebrates, offer a unique model for cartilage regeneration as their skeletal structure is entirely composed of cartilage due to the absence of endochondral ossification [70]. Col extracted from the skin of *Prionace glauca*, a commonly caught shark species, is easily obtainable and exhibits similarities to mammalian Col, making it a valuable resource for research and potential applications [71,72]. Diogo et al. utilized shark (*Prionace glauca*) skin Col for fabrication of 3D microporous cartilage engineering scaffolds [73]. The highly interconnected porous 3D constructs made of Col and HA (20:1) (Col-HA) were prepared with the cryogelation method. The metabolic activity of human adipose stem cells (hASCs) seeded on the Col-HA scaffolds was highly maintained with homogeneous distribution of viable cells on the scaffolds. A histological analysis indicated a significant increase in cell density from day 1 to day 7, suggesting cell proliferation and migration within the scaffolds. The cell-seeded scaffolds exhibited contraction, with both scaffold types being fully colonized by hASCs after 7 days. DNA quantification showed a gradual decrease in DNA content over 7 to 21 days, likely due to cell number reduction related to chondrogenic differentiation. The expression of chondrogenesis-related markers such as SOX-9, Col II, ACAN, and cartilage oligomeric matrix protein (COMP) was evaluated with a real-time quantitative polymerase chain reaction (qRT-PCR) and immunodetection, showing variations with scaffold type and culture conditions. Masson’s trichrome and alcian blue staining confirmed the presence of a cartilaginous extracellular matrix in the scaffolds, with stronger staining in chondrogenic culture conditions. Overall, it is demonstrated that the HA-Col scaffolds have excellent cytocompatibility, cell-mediated contraction, and support for chondrogenic differentiation of hASCs. While the results are promising, the contraction of the cell-seeded cryogels might affect pore size and cell migration, and future challenges are posed in understanding the effect of scaffolds’ cell-mediated contraction on chondrogenesis.

Jellyfish is one of the attractive options for a Col source. Jellyfish Col possesses high biocompatibility, low allergic response, and low potential for transmitting zoonotic diseases to humans [74]. Moreover, jellyfish can be extracted from the oceans with a positive carbon effect, and compared to higher trophic-level fish such as cod and tuna, they have a smaller food carbon footprint, indicating potential benefits for ocean health and reduced environmental impacts by incorporating more jellyfish into utilization [75,76]. Pugliano et al. synthesized a biocomposite of jellyfish-derived Col II, hMSCs, and TGF-β3 to fabricate therapeutic cartilage implants [77]. The Jellagen^®^ (Cardiff, United Kingdom) was utilized as marine collagen type II extracted from jellyfish, incorporated with hMSCs and TGF-β3 (Jellagen/TGF-β3 implants). Metabolic activity of hMSCs on Jellagen/TGF-β3 implants remained constant over the culture period, indicating the biocompatibility of the TGF-β3 nanoreservoir-enhanced implant. Indirect immunofluorescence confirmed the expression of chondrogenic markers SOX9, Runt-related transcription factor 2 (RUNX2), ACAN, and Col II, with higher Col II expression in the presence of TGF-β3 nanoreservoirs. Specific staining revealed increased GAG secretion in the Jellagen/TGF-β3 implant, visible through Safranin-O and alcian blue staining at 14 and 21 days, indicating enhanced chondrogenic differentiation potential. Alizarin staining was negative, indicating no osteogenic differentiation. Future study would need to enhance the in vivo delivery of active molecules such as TGF-β3 for cartilage repair, overcome the short half-life of growth factors, and improve cell attachment in 3D environments.

**Figure 5:**
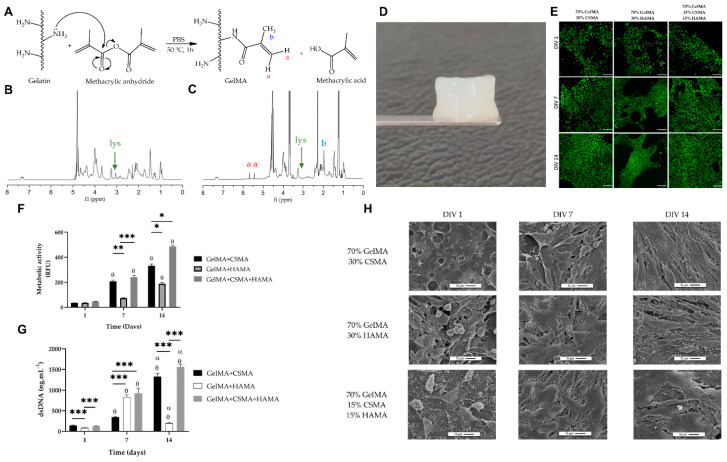
Marine proteins: GelMA-HAMA/CSMA hydrogel for cartilage tissue engineering. (**A**) Schematic diagram of reaction between gelatin and methacrylic anhydride. (**B**) Nuclear magnetic resonance (NMR) of Greenland-halibut-derived gelatin and (**C**) GelMA. The red a, blue b, and lys indicate chemical components of (**A**) and lysine, respectively. (**D**) Hydrogel of formulation of 70% GelMA, 15% CSMA, and 15% HAMA. (**E**) Live/dead assay for ATDC-5 cells seeded on the different GelMA-HAMA/CSMA hydrogels. The scale bars denote 100 µm. (**F**) Proliferation rate and (**G**) metabolic activity of ATDC5 cells for day 1 (θ) and 7 (α). The asterisks indicate significant differences between groups (* *p* < 0.05, ** *p* < 0.01, and *** *p* < 0.001). (**H**) SEM images of ATDC5 cells cultured on GelMA-HAMA/CSMA for 1, 7, and 14 days. Data reproduced from [82]. Copyrights MDPI 2023.

Gelatin, a protein derived from collagen, shares structural and functional properties with collagen, including high biocompatibility, biodegradability, low antigenicity, and the ability to promote cell attachment and growth [78]. Especially marine gelatin sourced from cold-water fish byproducts, which are often wasted in the fish processing industry, is gaining attention for its potential applications [79]. Various studies are exploring the use of fish byproducts to produce gelatins, offering a cost-effective and bioactive alternative with tunable physicochemical properties, thus addressing the challenge of waste valorization in the industry [80,81]. Machado et al. introduced marine gelatin from the skin of Greenland halibut as cell templates for cartilage tissue engineering, combined with methacrylated HA (HAMA) and methacrylated chondroitin sulfate (CSMA) (namely, GelMA-HAMA/CSMA hydrogel) to render a photocrosslinkable property [82] (Figure 5). Live/dead assays revealed that ATDC-5 cells remained viable for up to 14 days and were distributed well within all GelMA hydrogel compositions. Metabolic activity and proliferation of the cells increased over time in the culture, demonstrating the cytocompatibility of the GelMA-HAMA/CSMA hydrogel. A SEM analysis indicated that cells predominantly exhibited a rounded morphology, which is associated with a cartilage tissue phenotype. The results suggest that the prepared GelMA-HAMA/CSMA hydrogels can provide a suitable structural environment for chondroblast cells and hold potential for cartilage tissue engineering due to their biocompatibility and ability to support cell adhesion and growth, thus promoting chondrogenic differentiation. Fine-tuning of the UV irradiation process would be required to create a cytocompatible environment with high biocompatibility, promoting cell survival and maintaining their chondrogenic phenotype. Alves et al. utilized the exceptional transparency of GelMA hydrogel, which is enriched with ascorbic acid (AA) (GelMA-AA), for corneal stroma regeneration [80]. Marine gel was extracted from industrial wastes of Codfish (*Gadus morhua*) and incorporated with AA having an antioxidant character that could help keratocytes resist oxidative stress. When evaluating the stability, it was found that the degradation of GelMA hydrogels increased with the addition of AA, and the release of AA from the hydrogels followed a similar pattern. Enzymatic degradation using collagenase showed that the presence of AA affected the degradation rate, with higher concentrations leading to faster degradation. The optical properties of the GelMA-AA hydrogels remained consistent over time, with high light transmittance. When human keratocytes were encapsulated within the GelMA-AA hydrogels, live/dead assays showed that cells remained viable and spread throughout the hydrogels, with increased metabolic activity and cell elongation over time. Immunofluorescence staining indicated the expression of corneal ECM proteins and proteoglycans within the GelMA-AA hydrogels. The results suggested that GelMA-AA hydrogels can provide a suitable environment for human keratocytes, with their properties modulated by GelMA concentration and the presence of AA. Future challenges in exploiting GelMA-AA hydrogels for corneal stromal regeneration involve optimizing mechanical properties, stability, water retention, and cell viability, and managing the release of AA while maintaining mechanical integrity.

Fucoidan, a sulfated polysaccharide primarily extracted from brown seaweeds, consists mainly of sulfated fucose and various sugars, including uronic acids [83]. Due to its composition, it is often likened to sulfated glycosaminoglycans (GAGs) found in the extracellular matrix (ECM). Recent research has unveiled a wide range of biological activities associated with fucoidan, including its potential as an anticoagulant, antithrombotic, anti-inflammatory, anti-tumoral, antiviral, and antioxidant agent [40,84]. Carvalho et al. utilized Col-CS-fucoidan cryogel from marine sources to utilize as soft tissue cell culture scaffolds [85]. Each distinctive hydrogel was derived from marine sources: Col from jellyfish (Rhizostoma pulmo), CS from Squid (Dosidicus gigas), and fucoidan from brown algae (Fucus vesiculosus). Cytotoxicity testing was performed using L929 cells, and the results showed that extracts from the cryogels did not harm cell metabolic activity, confirming their non-toxic nature. Microscopy-based live/dead cell viability assays further validated the Col-CS-fucoidan cryogels’ biocompatibility. They revealed high cell viability, with predominantly live cells present on the cryogel surfaces, indicating a favorable microenvironment for cell growth. A SEM analysis revealed significant changes in the cryogel microstructure due to cell colonization. The original open porosity of the cryogels facilitated cell adhesion, proliferation, migration, and organization within the matrix. Cell morphology was assessed through fluorescence microscopy at different time points, confirming that the cells adhered to the cryogel matrices and acquired an elongated shape, indicating their complete integration into the scaffolds. These results demonstrated the Col-CS-fucoidan can serve as acellular biomaterials or as cell-laden cryogels for various tissue engineering applications including injectable therapies. In another study conducted by Carvalho et al., hASCs were laden in the Col-CS-fucoidan cryogels for cartilage tissue engineering applications [86]. Cell viability was assessed through fluorescence microscopy, which revealed a predominant presence of live cells with increasing culture time. This effect was more evident in the cryogels processed at −80 °C, possibly due to their internal network and pore size being more suitable for the hASC culture. The cells exhibited good adherence to the cryogels, confirming their adaptability to the matrices. Cell viability and proliferation were also assessed using the Alamar Blue assay and DNA quantification. Both assays indicated that the cryogels did not compromise hASC metabolic activity. Cell proliferation became more evident after day 3, reflecting the cells’ adaptability to the cryogel structures. Formulations that involved biopolymer blending, ionic bonding at −80 °C, and the inclusion of fucoidan provided a better environment for the cell culture compared to other conditions. These findings suggest that the marine Col-CS-fucoidan cryogels have the potential for successful tissue engineering applications, with −80 °C showing the most promising performance for cartilage regeneration. The study’s challenge lies in optimizing the formulation of marine Col-CS-fucoidan cryogels, focusing on factors including freezing temperatures and sulfation degree to achieve adequate mechanical properties and ionic bond density for cell culture applications. Future perspectives include testing these promising formulations in cell differentiation assays for chondrogenesis, assessing immunological responses, and conducting in vivo evaluations to understand their applicability in regenerating articular cartilage tissues.

In conclusion, the application of marine proteins in soft tissue engineering has been significantly notable, especially in diversifying sources due to limitations with traditional Col extraction from bovine and porcine origins. Marine organisms such as fish, sea urchins, jellyfish, sharks, starfish, and sponges present promising protein sources. These alternatives overcome dietary restrictions, religious constraints, and disease-related concerns seen in land-based sources. Moreover, utilizing discarded marine biomass promotes sustainability and minimizes environmental impact. Further research can focus on the in vivo efficacy and immunological responses of the marine-biomaterial-based engineered scaffolds, as well as optimizing biomechanical properties and stability. These will propel the field of soft tissue engineering using marine proteins, addressing current limitations and maximizing the potential of these novel marine biomaterials.

**Table 3:** Marine proteins in soft tissue engineering.

Engineered Forms	Application	Marine Biomaterials	Sources	Cells/Animal Tested	Key Findings	Ref.
Bilayered CA:L-CSPG nanofibrous sponge	Skin	Col	Stellate pufferfish (*Arothron stellatus)*	- NIH3T3 cell - HaCaT cell	CA:L-CSPG membranes exhibited excellent biocompatibility, antioxidant activity, and antibacterial activity with no hemolysis.	[68]
Freeze-dried FSC scaffold	Skin	Col	Grass carp (*Ctenopharyngodon idellus*)	L929 cell - New Zealand White rabbits	FSC promoted wound recovery in a burn wound with no scars.	[69]
Cryogelated HA-Col scaffold	Cartilage	Col	Shark skin (*Prionace glauca*)	hASC	HA-Col supports early chondrogenic differentiation, but external stimulation is needed for phenotype maintenance.	[73]
Freeze-dried Jellagen/TGF-β3 implants	Cartilage	Col	Jelly fish (*Rhizostoma pulmo*)	hMSCs	Incorporated TGF-β3 nanoreservoir in jellyfish collagen promoted up-regulation of chondrogenic markers of hMSCs.	[77]
GelMA-HAMA/CSMA hydrogel	Cartilage	Gel	Greenland halibut (*Reinhardtius hippoglossoides*)	ATDC-5 cell	The gelatin hydrogel provided cytocompatible environment to ATDC-5 cells.	[82]
GelMA/AA hydrogel	Cornea	Gel	Codfish (*Gadus morhua*)	Human KCs	AA in the GelMA facilitated Col production and corneal regeneration.	[80]
Col-CS-fucoidan cryogel	Skin	- Col - CS - Fucoidan	- Jellyfish (Rhizostoma pulmo) - Squid (Dosidicus gigas) - - Brown algae (Fucus vesiculosus)	L929 fibroblast	Marine Col-CS-fucoidan cryogels can be engineered as acellular biomaterials or cell-laden cryogels.	[85]
Col-CS-fucoidan cryogel	Cartilage	- Col - CS - Fucoidan	- Jellyfish (Rhizostoma pulmo) - Squid (Dosidicus gigas) - Brown algae (Fucus vesiculosus)	hASCs	Marine Col-CS-fucoidan cryogels showed excellent biomechanical properties and support behaviors of hASCs.	[86]

## 4. Conclusion and Future Perspectives

Marine-derived biomaterials hold great promise for biomedical applications due to their exceptional biocompatibility.

These materials offer numerous advantages over traditional mammalian sources, such as sustainability, scalability, reduced zoonotic disease risks, and fewer religious restrictions. Nonetheless, there are several challenges that must be addressed to promote their wider use in tissue engineering. One significant challenge, particularly in cold-water organisms, is the lower denaturation temperature of marine biomaterials. This affects processing conditions, requiring specialized facilities like cold rooms and modifications for thermal stability. A more sustainable approach involves exploring marine biomaterials from warm-water species with better thermal stability, allowing for gentler processing conditions and fewer modifications. Preservation of marine waste before extraction is another hurdle, especially on a larger scale. Rapid extraction of marine biomaterials after tissue harvest is crucial to prevent decomposition, driven by factors like bacteria, enzymatic autolysis, and lipid oxidation. Proper storage conditions are vital to maintain biomaterial integrity and prevent bacterial endotoxin contamination. Ensuring sterile conditions, following good manufacturing practices, and implementing rigorous quality control during extraction are of utmost importance, particularly at a commercial scale.

While in vitro investigations have provided valuable insights into the potential applications of marine biomaterials in tissue engineering, the transition to clinical evaluations remains a critical frontier. Given the possibility of allergens in certain marine species, rigorous quality control measures are imperative to ensure the complete removal of allergenic elements post-extraction. This underscores the need for meticulous examination of a patient’s medical history, particularly regarding seafood allergies, to preclude unforeseen reactions. To advance the field of marine-biomaterial-based tissue engineering, there is a pressing necessity for extensive research delving into the in vivo effects of each marine biomaterial derived from diverse marine origins. A comprehensive exploration of host immunological responses and the underlying mechanisms will be critical in unraveling the complex interactions of marine biomaterials within living organisms. Such in-depth studies will not only deepen our understanding of marine biomaterials but also pave the way for the widespread application of marine biomaterials in forthcoming translational applications.

## Figures and Tables

**Table 1:** Overview of marine biomaterials reported in this review.

Classification	Marine Biomaterials	Reported Marine Sources	Chemical Composition	Specific Features
Polysaccharides	Chitin	- Porifera	- Partially brominated α-chitin mineralized with silica and calcium carbonates	- Biocompatible, biodegradable, and non-toxicity - Antioxidant, antihypertensive, anticoagulant, anti-inflammatory, anticancer, antidiabetic, antimicrobial, and hypocholesterolemic activities
	Alginate	- Brown algae	Interconnected β-D-mannuronic (M) and α-L-guluronic (G) monomers	- Biocompatibility, low toxicity, low cost, and mild ion-mediated gelation
	Chitosan	- Mollusks - Crustaceans	Linear polysaccharide consisting of D-glucosamine and N-acetylglucosamine	- Abundance, biodegradability, biocompatibility, non-toxicity, and hydrophilicity - Antibacterial and anti-fungal properties with wound-healing effects
	κ-Carrageenan	- Red algae	Polysaccharide containing sulphate groups per disaccharide alternating G-, D-, or DA-units	- Immunomodulatory, anticoagulant, antioxidant, anticancer, and antiviral activity - Biodegradability, biocompatibility, high viscosity, and gelation ability
	Fucoidan	- Brown seaweed	Heteropolysaccharide with L-fucose-4-sulfate predominantly composed of extra L-fucose and sulphate groups	- Tailorable bioactivity with extraction process and the post-processing steps - Osteogenic property - Anticancer, antiviral, antiallergenic, anticoagulant, antioxidant, anti-inflammatory, immuno-modulatory, cardioprotective, and hepatoprotective effects
	Laminarin	- Brown algae	Branched polysaccharide consisting of (1–3)- β-d-glucan with β (1–6)-linkages/branching	- Antioxidant, anti-tumor, anticoagulant, anticancer, immunomodulatory, anti-obesity, antidiabetic, anti-inflammatory, wound-healing, and neuroprotective potential
	Ulvan	- Green seaweed	Sulfated polysaccharide of L-rhamnose, D-xylose, D-glucose, and D-glucuronic acid	- Immunomodulating, antiviral, antioxidant, antihyperlipidemic, and anticancer
	Agarose	- Red algae	Copolymer of β-1,3-linked d-galactose and α-1,4-linked 3,6-anhydro-α-l-galactose residues	- Self-gelling, water-solubility, adjustable mechanical properties - Non-immunogenic properties and cytocompatibility
	GY785	- Deep-sea bacteria	Sulfated nonasaccharide repeating unit with glucose, galactose, glucuronic acid, and galacturonic acid	- Anticoagulant properties, chondrogenic property, anticancer effects
Proteins	Collagen	- Stellate pufferfish - Grass carp - Shark - Jelly fish	Repeating sequences of glycine–proline–hydroxyproline amino acid triplets	- Biodegradability, weak antigenicity, and superior biocompatibility
	Gelatin	- Greenland halibut - Codfish	Hydrolyzed collagen	- Cheap and readily available with high biocompatibility and biodegradability - Cell recognition by RGD peptide and diverse functional groups

## Data Availability

No new data were created or analyzed in this study. Data sharing is not applicable to this article.
